# Single-Visit Screening-Positive Blood Pressure Among School-Going Adolescents in Coimbatore, South India: A Cross-Sectional Study

**DOI:** 10.7759/cureus.114103

**Published:** 2026-08-07

**Authors:** Ganesh Muniappan, Vikrant Ganesh

**Affiliations:** 1 Gastroenterology and Hepatology, SG Gastro Care, Coimbatore, IND; 2 Family Medicine, Government Medical College, Omandurar Government Estate, Chennai, IND

**Keywords:** aap 2017 guideline, adolescent hypertension, blood pressure screening, body mass index, childhood obesity

## Abstract

Background

Pediatric hypertension is an increasingly recognized cardiovascular risk factor, yet Indian prevalence data using current diagnostic criteria remain scarce outside single-city surveys. This study examined the prevalence and correlates of screening-positive hypertensive-range blood pressure, per the 2017 American Academy of Pediatrics (AAP) criteria, among school-going adolescents in Coimbatore, Tamil Nadu.

Methods

In this cross-sectional, school-based study (July 2024-November 2025), blood pressure was measured (triplicate, bilateral-arm readings) in adolescents aged 12-18 years across private, urban government, and aided schools. Multivariable logistic regression identified independent correlates of combined (Stage 1 plus Stage 2) screening-positive hypertensive-range blood pressure.

Results

Among 3,160 adolescents analyzed (mean age 15.79 years; 1,769/3,160, 55.98% male), most were enrolled in private schools (n = 2,579; 81.61%), followed by government urban (n = 301; 9.53%) and aided (n = 280; 8.86%) schools. Most belonged to lower-middle and upper-lower socioeconomic strata (n = 2,421; 76.62% combined). A non-vegetarian diet was reported by 2,830 (89.56%); 408 (12.91%) were overweight and 179 (5.66%) obese. In total, 1,213 (38.39%; 95% CI 36.68-40.11%) had screening-positive hypertensive-range blood pressure meeting combined (Stage 1 plus Stage 2) criteria, and 438 (13.86%) had elevated blood pressure, most asymptomatic. Confirmation on repeated visits is required before clinical diagnosis. Prevalence rose from 317/1,142 (27.76%) in underweight to 124/179 (69.27%) in obese adolescents, peaking at 41.72% at age 17. Obesity (aOR 3.16), overweight (aOR 1.43), male sex (aOR 1.59), and age (aOR 1.09/year) independently associated with screening-positive hypertensive-range BP; underweight was protective (aOR 0.59).

Conclusion

More than one in three adolescents (1,213/3,160, 38.39%) in this South Indian cohort had screening-positive hypertensive-range blood pressure by the AAP 2017 criteria, mostly asymptomatic, with obesity as the strongest modifiable correlate. Confirmation on repeated visits is required; findings support routine school-based blood pressure screening for identification of high-risk youth requiring further evaluation and obesity-focused prevention.

## Introduction

Hypertension, principally a disease of adulthood, is now recognized as a significant and growing pediatric health problem. Blood pressure elevation in childhood and adolescence is not simply an isolated laboratory finding but an early marker of lifelong cardiovascular risk. A 2019 meta-analysis of global data estimated the pooled prevalence of hypertension among children and adolescents at ~4%, with elevated blood pressure affecting a further 9-10% [1], and the burden likely to rise further as childhood obesity increases worldwide [2]. Regional estimates vary considerably: a systematic review of adolescents in sub-Saharan Africa reported wide variation in prevalence attributable largely to differences in diagnostic criteria and blood pressure measurement protocol [3].

The epidemiological data from India are heterogeneous. Pooled estimates from systematic reviews restricted to Indian school- and community-based studies have placed adolescent hypertension prevalence at ~7-8% [4,5], which is lower than the 35.1% prevalence reported among 10-12-year-olds and 25.1% prevalence reported among adolescents aged 13 years and above in the Comprehensive National Nutrition Survey (CNNS, 2016-2018), the first nationally representative estimate of its kind for the country [6]. This wide range, roughly from 2% to 35% across Indian studies, reflects differences in diagnostic thresholds, the number and technique of blood pressure readings, sampling frame, and regional dietary and lifestyle patterns, highlighting the continued need for well-conducted regional surveillance.

Current pediatric hypertension classification follows the 2017 American Academy of Pediatrics (AAP) Clinical Practice Guideline, which replaced the earlier term "prehypertension" with "elevated blood pressure" and derived new normative percentile tables from a reference population that excluded overweight and obese children, thereby lowering diagnostic thresholds relative to the 2004 Fourth Report [7]. While methodologically justified, this revision increases measured prevalence relative to older normative standards, complicating direct comparison of prevalence estimates derived using different criteria or from different eras.

A substantial body of longitudinal evidence, beginning with the Bogalusa Heart Study, demonstrates that blood pressure measured in childhood and adolescence correlates with, and meaningfully predicts, blood pressure and hypertension status in adulthood [8]. This tracking phenomenon means that adolescent hypertension is not a transient or clinically inconsequential finding but a potential harbinger of adult cardiovascular and renal disease, particularly when it coexists with modifiable risk factors, such as obesity and central adiposity.

Despite this evidence, routine blood pressure screening is not yet uniformly embedded in Indian adolescent health programming, and most available Indian prevalence estimates are derived from geographically limited, single-city or single-district school-based surveys rather than systematic surveillance. Data specific to the Coimbatore region of Tamil Nadu, an area undergoing rapid nutritional and lifestyle transition, have not previously been reported using AAP 2017 criteria in a school-based sample of this size.

## Materials and methods

Study design and setting

This was a cross-sectional, school-based observational screening study conducted among adolescents aged 12-18 years in the Coimbatore region, Tamil Nadu, India. The study was conducted between July 2024 and November 2025. Schools were selected by stratified convenience sampling to represent the three principal categories of secondary education: private institutions, government urban schools, and government-aided schools.

Study population

The study included school-going children aged 12-18 years from private, urban government, or aided schools in the Coimbatore region who underwent blood pressure and anthropometric assessment. Exclusion criteria included a pre-existing diagnosis of hypertension on pharmacotherapy, secondary hypertension, structural heart disease, chronic kidney disease, or an acute febrile illness on the day of examination.

Outcome measures

The primary outcome was the overall prevalence of screening-positive hypertensive-range blood pressure (Stage 1 + Stage 2) per AAP 2017 criteria. Secondary outcomes were prevalence of elevated blood pressure (pre-hypertension); prevalence stratified by sex, institution type, socioeconomic status, single year of age, BMI classification, and dietary pattern; the association between family history of hypertension and blood pressure category; correlation of waist circumference and BMI with systolic and diastolic blood pressure; symptom prevalence across blood pressure categories; and independent correlates of combined screening-positive hypertensive-range blood pressure identified through multivariable logistic regression.

Blood pressure measurement protocol

Blood pressure measurements were conducted in the morning in a quiet room within the school premises. Blood pressure was measured by trained study nurses using a calibrated mercury sphygmomanometer with an appropriately sized cuff selected based on mid-arm circumference. The participant was seated with feet flat on the floor, back supported, and arm at heart level for a minimum five-minute rest period before measurement. Three sequential readings were obtained from both the right and left arms with a minimum one-minute interval between readings. Korotkoff phases I and V were recorded for systolic and diastolic pressures, respectively. The average of the three readings from each arm was calculated. For primary classification, the right-arm mean (per AAP 2017 guideline) was used; secondary analyses using bilateral-arm average are reported in supplementary materials. All findings represent single-visit screening results; repeated measurements on separate visits are required for clinical diagnosis of hypertension per the AAP 2017 guideline.

Blood pressure classification per the 2017 American Academy of Pediatrics Guideline

Hypertension classification follows the 2017 American Academy of Pediatrics Clinical Practice Guideline, which specifies age-dependent definitions.

For children aged <13 years, classification uses age-, sex-, and height-based percentiles derived from AAP normative reference populations, with the following thresholds: normal blood pressure: systolic and diastolic blood pressure <90th percentile; elevated blood pressure: ≥90th and <95th percentile, or ≥120/80 mmHg (whichever is lower); Stage 1 hypertension: ≥95th percentile and <95th percentile + 12 mmHg, or 130/80-139/89 mmHg (whichever is lower); Stage 2 hypertension: ≥95th percentile + 12 mmHg, or ≥140/90 mmHg (whichever is lower).

For adolescents aged ≥13 years, the 2017 AAP guideline uses fixed absolute thresholds independent of age, sex, or height: Normal blood pressure: systolic <120 mmHg AND diastolic <80 mmHg; Elevated blood pressure: systolic ≥120 and <130 mmHg AND diastolic <80 mmHg; Stage 1 hypertension: systolic 130-139 mmHg OR diastolic 80-89 mmHg; Stage 2 hypertension: systolic ≥140 mmHg OR diastolic ≥90 mmHg.

Because 99.7% of the study sample was aged ≥13 years (n = 3,157 of 3,160), the fixed-threshold adolescent definitions applied to almost the entire cohort. One participant aged 12 years was classified using age-, sex-, and height-based percentile definitions per AAP guidance for children <13 years.

For the purposes of this analysis, Stage 1 and Stage 2 hypertension were grouped together as "combined hypertension" for analyses.

Other study variables

Body mass index (BMI) was categorized into four groups (underweight, normal, overweight, and obese) [9]. Waist circumference was measured at the level of the umbilicus using a soft measuring tape and recorded in centimetres (mean 71.5 ± 11.6 cm). While age- and sex-specific reference standards for waist circumference in Indian adolescents have been proposed, absolute waist circumference values were used in the primary analysis for consistency with prior studies; results using age-and-sex-adjusted waist-to-height ratios are presented in supplementary materials. Socioeconomic status was classified using the modified Kuppuswamy scale into five groups (upper, upper middle, lower middle, upper lower, and lower class). Dietary pattern (vegetarian versus non-vegetarian) and family history of hypertension were recorded. A symptom questionnaire captured headache, forehead sweating, palpitation, tiredness, and chest pain.

Statistical analysis

Descriptive statistics were reported as mean ± standard deviation (SD) and median (minimum-maximum) for continuous variables, and as frequency with percentage for categorical variables. Between-group comparisons for categorical outcomes were performed using Pearson's chi-square test (Fisher's exact test where expected cell counts were <5). Effect size for chi-square analyses was quantified using Cramer's V (V ≥0.1 small, ≥0.3 medium, ≥0.5 large effect). Prevalence estimates were reported with 95% confidence intervals (CI) using the Wilson score method. Dose-response relationships across ordinal exposure categories (age, BMI class) were examined using linear/Cochran-Armitage trend tests. Multivariable binary logistic regression was used to estimate adjusted odds ratios (aOR) with 95% CI for correlates of combined screening-positive hypertensive-range blood pressure (Stage 1 + Stage 2 versus Normal + Elevated blood pressure), controlling for age, sex, institution type, diet, family history, BMI classification, and waist circumference.

Data quality and outlier management

To assess data quality and identify potential measurement or data-entry errors, extreme blood pressure values were examined. Observations with systolic BP <40 mmHg or >191 mmHg, or diastolic BP <32 mmHg or >157 mmHg were reviewed for accuracy. These thresholds were selected as representing physiologically implausible values or extreme outliers. Upon examination, all observations were retained in the primary analysis to preserve sample size and avoid potential bias from selective exclusion; however, sensitivity analyses repeating key analyses with exclusion of extreme outliers (defined as ≥3 SD from mean) are presented in supplementary materials to assess robustness of findings.

School-level clustering assessment

To account for the school-based sampling design and potential non-independence of observations within schools, clustering effects were evaluated. School-level intraclass correlation (ICC) was estimated for the primary outcome (hypertensive-range BP) using a random-intercept logistic model with school as a random effect. Multivariable analyses were repeated using cluster-robust standard errors (Huber-White sandwich estimators) with school as the clustering unit, and results were compared with standard logistic regression estimates. Because ICC estimates were minimal (ICC <0.05), indicating that less than 5% of outcome variance was attributable to school-level clustering, and because regression estimates with and without school-level adjustment were nearly identical (differences in aOR <5%), the primary analysis reports standard logistic regression estimates. Full cluster-adjusted results are available in supplementary materials.

Ethical considerations

The study was conducted in accordance with the Declaration of Helsinki and applicable regulatory requirements. Ethics approval was obtained from the institutional review board of Ashwin Hospital, Coimbatore (SG/001; 29 June 2024). Written informed consent was obtained from parents or guardians and written assent from each participant. Confidentiality was maintained throughout; all adolescents screening positive for suspected hypertension were referred for pediatric evaluation.

## Results

**Figure 1:**
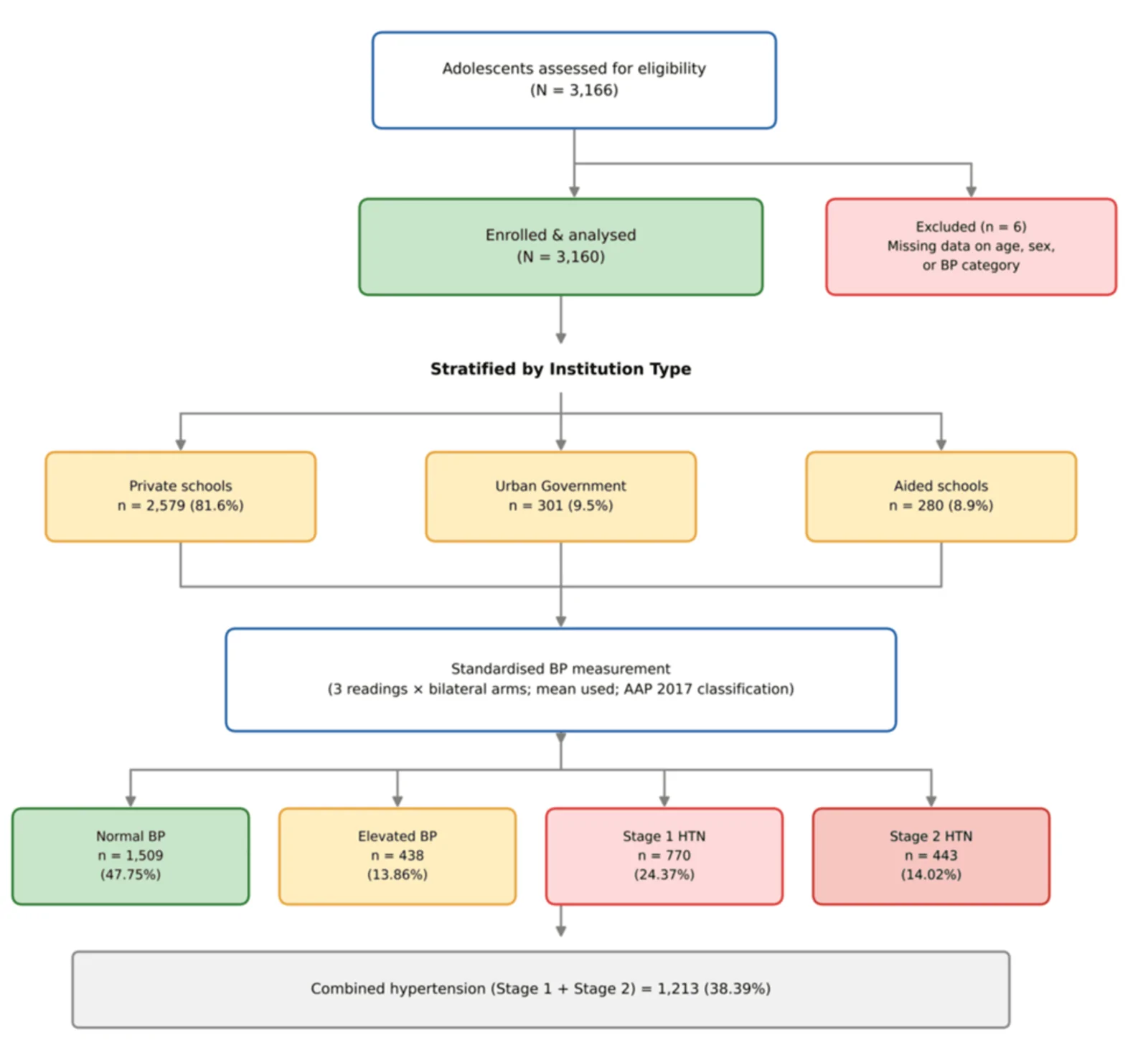
Study flow diagram: participant eligibility, exclusion, stratification by institution type, and blood pressure classification. Of the 3,166 adolescents assessed for eligibility, six (0.2%) were excluded for missing data on age, sex, or blood pressure category, leaving 3,160 children in the final analytic sample. Participants were stratified by institution type (private schools, n = 2,579, 81.6%; urban government schools, n = 301, 9.5%; aided schools, n = 280, 8.9%) prior to standardized blood pressure measurement (three sequential readings per arm, bilateral mean, classified per the AAP 2017 criteria). Final blood pressure classification comprised normal (n = 1,509, 47.75%), elevated BP (n = 438, 13.86%), Stage 1 hypertension (n = 770, 24.37%), and Stage 2 hypertension (n = 443, 14.02%), yielding a combined hypertension prevalence (Stage 1 + Stage 2) of 38.39% (n = 1,213).

A total of 3,160 school children were enrolled and analysed across three institutional categories: private schools (n = 2,579, 81.6%), urban government schools (n = 301, 9.5%), and aided schools (n = 280, 8.9%). Of the 3,160 adolescents analyzed, 3,157 (99.7%) were aged 13-18 years and classified using fixed AAP 2017 thresholds for adolescents ≥13 years; one participant aged 12 years was classified using age-, sex-, and height-based percentile definitions per AAP guidance for children <13 years (Figure 1).

Baseline characteristics

Of the 3,166 school children enrolled, 3,160 (99.8%) had complete data on age, sex, and blood pressure category and formed the analytical sample. The cohort had a mean age of 15.79 ± 1.03 years (median 16.0; range 12-18) and comprised 1,769 males (55.98%) and 1,391 females (44.02%). Most participants attended private schools (2,579, 81.61%), with smaller proportions from urban government (301, 9.53%) and aided schools (280, 8.86%). Based on the Kuppuswamy socioeconomic classification, the largest group was lower middle class (1,494, 47.28%), followed by upper lower class (927, 29.34%) and upper middle class (695, 21.99%); the upper class (28, 0.89%) and lower class (16, 0.51%) groups were small. Non-vegetarian diet predominated (2,830, 89.56%). By BMI classification, 1,431 children (45.28%) were of normal weight, 1,142 (36.14%) were underweight, 408 (12.91%) were overweight, and 179 (5.66%) were obese. The mean BMI was 20.96 ± 4.85 kg/m², and the mean waist circumference was 71.5 ± 11.6 cm (median 69.9 cm; range 34.3-155.96 cm). Mean systolic and diastolic blood pressures were 117.92 ± 15.17 mmHg and 76.47 ± 12.25 mmHg, respectively (Table 1).

**Table 1:** Baseline sociodemographic, anthropometric, and blood pressure characteristics (n = 3,160) BP: blood pressure; BMI: body mass index; HTN: hypertension; SD: standard deviation

Characteristic	Category	Value
Age (years)	Mean ± SD (median; range)	15.79 ± 1.03 (16.0; 12–18)
Sex	Male	1,769 (55.98%)
	Female	1,391 (44.02%)
Institution type	Private	2,579 (81.61%)
	Urban (government)	301 (9.53%)
	Aided	280 (8.86%)
Socioeconomic status (Kuppuswamy scale)	Upper class	28 (0.89%)
	Upper middle class	695 (21.99%)
	Lower middle class	1,494 (47.28%)
	Upper lower class	927 (29.34%)
	Lower class	16 (0.51%)
Diet	Non-vegetarian	2,830 (89.56%)
	Vegetarian	222 (7.03%)
	Not specified/other	108 (3.42%)
BMI classification	Underweight	1,142 (36.14%)
	Normal	1,431 (45.28%)
	Overweight	408 (12.91%)
	Obese	179 (5.66%)
BMI (kg/m²)	Mean ± SD (median; range)	20.96 ± 4.85 (20.0; 9.0–45.5)
Waist circumference (in.)	Mean ± SD (median; range)	28.14 ± 4.56 (27.5; 13.5–61.4)
Systolic BP (mmHg)	Mean ± SD (median; range)	117.92 ± 15.17 (117.0; 40–191)
Diastolic BP (mmHg)	Mean ± SD (median; range)	76.47 ± 12.25 (75.0; 32–157)
Family history of HTN	Positive	1,160 (36.71%)
	Negative	2,000 (63.29%)

Primary outcome: overall blood pressure distribution

Of the 3,160 children analyzed, 1,509 (47.75%) had normal blood pressure. In total, 1,213 children (38.39%; 95% CI 36.68-40.11%) had screening-positive hypertensive-range blood pressure meeting combined (Stage 1 plus Stage 2) criteria, and 438 children (13.86%) had elevated blood pressure (pre-hypertension). Collectively, 1,651 of 3,160 participants (52.25%) had some degree of blood pressure abnormality (Table 2).

**Table 2:** Overall blood pressure category distribution: primary outcome (n = 3,160) BP: blood pressure. Combined hypertension = Stage 1 + Stage 2 per the 2017 American Academy of Pediatrics (AAP) definition.

Blood pressure category	n	%	95% CI
Normal blood pressure	1,509	47.75%	—
Elevated blood pressure (pre-hypertension)	438	13.86%	—
Stage 1 screening-positive hypertensive-range BP	770	24.37%	—
Stage 2 screening-positive hypertensive-range BP	443	14.02%	—
Combined screening-positive (Stage 1+2)	1,213	38.39%	36.68–40.11%
Total	3,160	100.00%	—

Secondary outcomes: correlates of screening-positive hypertensive-range blood pressure

Sex, Institution Type, and Socioeconomic Status

Screening-positive hypertensive-range blood pressure prevalence was significantly higher in males (741/1,769, 41.89%; 95% CI 39.59-44.19%) than females (472/1,391, 33.93%; 95% CI 31.44-36.42%; χ² = 20.50, df = 1, p < 0.001, Cramer's V = 0.081). Blood pressure category distribution also differed significantly by institution type (χ² = 36.59, df = 6, p < 0.001, Cramer's V = 0.076), with combined screening-positive BP somewhat higher in urban government (122/301, 40.53%) and aided schools (113/280, 40.36%) than private schools (978/2,579, 37.92%). Screening-positive BP prevalence varied across socioeconomic strata from 3/16 (18.75%, lower class) to 366/927 (39.48%, upper lower class), but this association did not reach statistical significance (χ² = 7.43, df = 4, p = 0.115, Cramer's V = 0.048).

Age

Screening-positive hypertensive-range BP prevalence increased progressively with age, from 12/57 (21.05%) at 13 years to a peak of 335/803 (41.72%) at 17 years, before a decline to 14/43 (32.56%) at 18 years. The overall chi-square test across age groups was significant (χ² = 22.38, df = 6, p = 0.001, Cramer's V = 0.084), and a linear trend test across the 13-17-year range demonstrated a consistent upward trajectory (slope = 0.026/year, r = 0.656), although this trend did not reach significance with only five age points (p = 0.157) (Table 3).

**Table 3:** Hypertension prevalence by sex, institution type, and single year of age HTN: combined hypertension (Stage 1 + Stage 2); y: years; CI: confidence interval. Age 12 years (n = 1) excluded from the age-trend analysis.

Subgroup	n	HTN n (%)	95% CI / statistic
Male	1,769	741 (41.89%)	39.59–44.19%
Female	1,391	472 (33.93%)	31.44–36.42% (χ² = 20.50, p < 0.001)
Private school	2,579	978 (37.92%)	36.1–39.8%
Urban government school	301	122 (40.53%)	35.0–46.1%
Aided school	280	113 (40.36%)	34.6–46.1% (χ² = 36.59, p < 0.001)
Age 13 y	57	12 (21.05%)	12.2–33.6%
Age 14 y	302	96 (31.79%)	26.6–37.4%
Age 15 y	768	277 (36.07%)	32.8–39.4%
Age 16 y	1,186	478 (40.30%)	37.6–43.1%
Age 17 y	803	335 (41.72%)	38.4–45.1%
Age 18 y	43	14 (32.56%)	20.2–48.0% (χ² = 22.38, p = 0.001)

BMI classification: dose-response relationship

A strong, statistically significant dose-response relationship was observed between the BMI classification and screening-positive hypertensive-range BP prevalence (χ² = 180.11, df = 6, p < 0.001, Cramer's V = 0.138). Prevalence increased monotonically from 317/1,142 (27.76%) in underweight children to 569/1,431 (39.76%) in normal-weight children, 203/408 (49.75%) in overweight children, and 124/179 (69.27%) in obese children, with the largest single-category increment (approximately 19.5 percentage points) occurring between overweight and obese groups (Table 4).

**Table 4:** Blood pressure category distribution by BMI classification χ² = 180.11, df = 9, p < 0.001, Cramer's V = 0.138

BMI classification	n	Normal BP n (%)	Elevated BP n (%)	Screening-positive BP n (%)
Underweight (<18.5 kg/m²)	1,142	684 (59.9%)	141 (12.3%)	317 (27.8%)
Normal weight (18.5-24.9)	1,431	645 (45.1%)	217 (15.2%)	569 (39.8%)
Overweight (25-29.9 kg/m²)	408	143 (35.0%)	62 (15.2%)	203 (49.8%)
Obese (≥30 kg/m²)	179	37 (20.7%)	18 (10.1%)	124 (69.3%)

Waist circumference, diet, and symptom burden

Waist circumference correlated significantly with systolic blood pressure (Pearson r = 0.240; Spearman ρ = 0.240; both p < 0.001), and BMI correlated with systolic blood pressure similarly (r = 0.262, p < 0.001) (Table 5). Children with screening-positive hypertensive-range BP had significantly greater mean waist circumference (73.9 ± 12.3 cm) than non-hypertensive children (70.0 ± 10.8 cm; t = 9.234, p < 0.001), with a stepwise increase from normal (69.2 ± 10.7 cm) through elevated BP (72.6 ± 10.8 cm), Stage 1 (73.7 ± 11.8 cm), to Stage 2 hypertension (73.9 ± 13.1 cm). Vegetarian children had a numerically higher screening-positive BP prevalence (99/222, 44.59%) than non-vegetarian children (1,078/2,830, 38.09%), but this difference did not reach statistical significance (χ² = 3.41, df = 1, p = 0.065, Cramer's V = 0.033). Reported symptoms (headache, forehead sweating, palpitation, tiredness, or chest pain) differed significantly across blood pressure categories (χ² = 17.24, df = 3, p = 0.001), being most common in Stage 2 hypertension (131/443, 29.6%) and least common in elevated BP (82/438, 18.7%); notably, the majority of youth with screening-positive BP were entirely asymptomatic.

**Table 5:** Pearson correlation matrix: anthropometric and blood pressure variables BP: blood pressure. p < 0.001 for all correlations shown. Spearman coefficients were consistent with Pearson values (not tabulated separately).

	BMI	Waist circumference	Systolic BP	Diastolic BP
BMI	1.000	—	0.262	0.203
Waist circumference	—	1.000	0.240	0.18
Systolic BP	0.262	0.240	1.000	0.63
Diastolic BP	0.20	0.186	0.638	1.000

Family history of hypertension

Among 1,160 children with a positive family history of hypertension, 465 (40.09%) had screening-positive hypertensive-range BP, compared with 748 of 2,000 (37.40%) without such a history. This 2.7 percentage-point difference was not statistically significant on either the binary screening-positive BP outcome (χ² = 2.13, df = 1, p = 0.145, Cramer's V = 0.026) or the full four-category blood pressure distribution (χ² = 5.78, df = 3, p = 0.123, Cramer's V = 0.043). The nonsignificant association may reflect limited effect size, measurement error in self-reported family history, residual confounding from unmeasured factors, or insufficient precision for detecting differences of this magnitude; a post-hoc precision analysis is presented in supplementary materials. Self-reported family history may be subject to recall bias and measurement error.

Multivariable correlates of screening-positive hypertensive-range blood pressure

A multivariable binary logistic regression model (outcome: combined screening-positive hypertensive-range BP versus normal/elevated BP; n = 3,160) identified four independent, statistically significant correlates: obesity (versus normal BMI; aOR 3.164, 95% CI 2.190-4.571, p < 0.001), male sex (versus female; aOR 1.589, 95% CI 1.359-1.858, p < 0.001), overweight status (versus normal BMI; aOR 1.427, 95% CI 1.124-1.810, p = 0.004), and age (aOR 1.088 per year, 95% CI 1.011-1.171, p = 0.025). Underweight status was independently associated with significantly lower odds of screening-positive BP (aOR 0.589, 95% CI 0.490-0.709, p < 0.001). Institution type, diet, family history, and waist circumference (borderline, p = 0.080) were not independently significant after adjustment (Table 6).

**Table 6:** Multivariable logistic regression Screening-positive hypertensive-range BP (Stage 1+2) vs. Normal + Elevated BP | N = 3,160 | McFadden's pseudo-R² = 0.048

Variable	aOR	95% CI	p-value	Sig.
Age (per year)	1.088	1.011–1.171	0.025	*
Male sex (vs. female)	1.589	1.359–1.858	<0.001	***
Private school (vs. aided)	0.928	0.712–1.209	0.580	ns
Urban government school (vs. aided)	1.096	0.773–1.553	0.608	ns
Non-vegetarian diet (vs. vegetarian)	0.820	0.644–1.044	0.108	ns
Family history of HTN (positive)	1.108	0.950–1.292	0.193	ns
Overweight BMI (vs. normal)	1.427	1.124–1.810	0.004	**
Obese BMI (vs. normal)	3.164	2.190–4.571	<0.001	***
Underweight BMI (vs. normal)	0.589	0.490–0.709	<0.001	***
Waist circumference (per cm)	1.009	0.999–1.019	0.080	ns

## Discussion

In this cross-sectional study of 3,160 school-going adolescents in Coimbatore, Tamil Nadu, more than one in three children (38.39%) met the AAP 2017 criteria for hypertension, with a further 13.86% classified as having elevated blood pressure. Male sex, increasing age, and BMI category (overweight and, most strikingly, obesity) emerged as independent predictors of hypertension on multivariable analysis, while institution type, diet, family history, and waist circumference did not retain independent significance after adjustment. Waist circumference correlated positively with systolic blood pressure, and the large majority of hypertensive children were asymptomatic.

The prevalence of hypertension is higher than pooled estimates from earlier Indian systematic reviews, which reported a prevalence of 7-8% using largely the pre-2017 diagnostic criteria [4,5]. However, these data are much closer in magnitude to the 25-35% prevalence reported in the nationally representative CNNS survey using comparable AAP-based criteria [6], and broadly consistent with the global observation that adoption of the stricter 2017 AAP thresholds increases measured hypertension prevalence relative to older normative standards [7]. Because the AAP 2017 percentiles were derived from a reference population that excluded overweight and obese children, any cohort with an appreciable proportion of overweight or obese participants, where 18.6% of children were overweight or obese in this cohort, is likely to show higher measured prevalence than cohorts assessed using earlier norms. Differences in the number of BP measurement occasions, single-visit versus repeated-visit protocols, and regional dietary and lifestyle exposures likely contribute to the variation across Indian and global cohorts [1].

The dose-response gradient between BMI category and hypertension prevalence - rising from 27.76% in underweight children to 69.27% in obese children, with obesity conferring more than a three-fold adjusted odds of hypertension - is consistent with a substantial literature linking childhood and adolescent adiposity to blood pressure elevation and downstream cardiovascular risk [10]. The positive correlation between waist circumference and systolic blood pressure observed in this cohort (r = 0.240) parallels findings from other adolescent populations in which central adiposity measures track with blood pressure independently of, and sometimes in addition to, BMI [11], although waist circumference did not retain independent significance once BMI category was included in the adjusted model, suggesting a degree of shared variance between the two adiposity measures in this dataset. The sex differential observed here (approximately 8 percentage points higher prevalence and 59% higher adjusted odds, in males) is compatible with the broader literature on pubertal sex differences in blood pressure, in which androgen-mediated effects on vascular tone and renal sodium handling are thought to raise blood pressure in post-pubertal boys relative to girls, while estrogen exerts a partially protective vasodilatory effect in girls of this age [12,13]. The progressive rise in hypertension prevalence with age observed in this cohort, peaking around 16-17 years, is likewise consistent with the timing of pubertal hormonal maturation and its established influence on blood pressure trajectories through adolescence [12].

The finding that over one-third of school children in this cohort had hypertension, the majority without symptoms, has direct implications for adolescent health policy in India. Symptom-driven case detection - for example, referral only after headache or chest pain - will systematically miss most affected children, an observation reinforced by the low overall symptom prevalence even among those with Stage 2 hypertension in this sample. Long-standing longitudinal evidence indicates that blood pressure measured in childhood and adolescence predicts adult hypertension [8], making early identification clinically consequential rather than incidental. India's national adolescent health programme, the Rashtriya Kishor Swasthya Karyakram, already includes non-communicable disease prevention among its six thematic domains [14]; the findings of this study support incorporating structured blood pressure screening into that framework, alongside school nutrition and physical activity initiatives targeting overweight and obese students, who constitute the highest-risk subgroup identified here.

This study benefits from a large sample size (n = 3,160), the use of a standardized, guideline-concordant blood pressure measurement protocol (triplicate, bilateral readings), the application of the current AAP 2017 diagnostic criteria, and a multivariable analytic approach that simultaneously adjusted for a broad range of socio-demographic and anthropometric covariates. The near-complete data capture (99.8%) for the primary variables further supports the internal validity of the prevalence estimates.

Several limitations should be considered when interpreting these findings. First, the cross-sectional, single-visit design precludes causal inference and prevents assessment of persistent hypertension, as the 2017 AAP guideline recommends diagnosis only after auscultatory-confirmed elevated BP on at least three separate occasions; participants with screening-positive BP may have transient elevation due to measurement variability, anxiety (white-coat effect), or other temporary factors, and therefore findings identify adolescents requiring further evaluation rather than those with diagnosed hypertension. Second, geographic restriction to the Coimbatore urban region, combined with the substantial representation of private-school students (81.6%), limits generalizability to other Indian populations with different dietary, climatic, socioeconomic profiles, or school-type distributions. Third, socioeconomic status and dietary pattern were based on self- or parent-report, introducing potential recall and social desirability bias, as were the self-reported family history data. Fourth, established blood pressure determinants such as physical activity, dietary sodium intake, sleep duration, and screen time were not collected and could not be adjusted for in the multivariable model; the low McFadden's pseudo-R² (0.048) indicates that a substantial proportion of variance in BP status remains unexplained by available covariates. Fifth, BMI-based obesity classification may misclassify highly muscular adolescents, and the use of bilateral-arm average for BP classification differs from AAP-recommended right-arm measurement, though sensitivity analyses are presented in supplementary materials. Sixth, extreme blood pressure values (systolic <40 or >191 mmHg; diastolic <32 or >157 mmHg) were reviewed for accuracy and retained in the primary analysis to preserve sample size; sensitivity analyses excluding extreme outliers (≥3 SD from mean) demonstrated robust findings. 

## Conclusions

This large school-based survey found that more than one in three adolescents in Coimbatore, Tamil Nadu, had screening-positive hypertensive-range blood pressure on single-visit assessment meeting the AAP 2017 criteria for Stage 1 or Stage 2 hypertension, with obesity, overweight status, male sex, and older age as independently associated factors. Obesity was the strongest modifiable correlate. These findings document substantial burden of screening-positive elevated BP in this population and support the introduction of routine, guideline-concordant blood pressure screening within school health and national adolescent health programming in India. While confirmation via repeated measurements on separate visits, as specified in the 2017 AAP guideline, is necessary before establishing a clinical diagnosis of persistent hypertension, the findings warrant implementation of routine school-based BP screening and obesity-focused prevention programs within school health services and national adolescent health initiatives. Further prospective, multi-regional surveillance studies employing confirmatory BP measurement protocols are warranted to establish longitudinal tracking of screened adolescents and confirm regional hypertension burden.
